# Role of Methoprene-tolerant in the regulation of oogenesis in *Dipetalogaster maxima*

**DOI:** 10.1038/s41598-022-18384-5

**Published:** 2022-08-20

**Authors:** Fabian O. Ramos, Marcela Nouzova, Leonardo L. Fruttero, Jimena Leyria, Rodrigo Ligabue-Braun, Fernando G. Noriega, Lilian E. Canavoso

**Affiliations:** 1 Departamento de Bioquímica Clínica, Facultad de Ciencias Químicas, Universidad Nacional de Córdoba, Córdoba, Argentina; 2 Centro de Investigaciones en Bioquímica Clínica e Inmunología (CIBICI), Consejo Nacional de Investigaciones Científicas y Técnicas (CONICET), Córdoba, Argentina; 3 Department of Biological Sciences and Biomolecular Science Institute, Florida International University, Miami, FL USA; 4 Institute of Parasitology, Biology Centre CAS, Ceske Budejovice, Czech Republic; 5 Department of Pharmacosciences and Graduate Program in Biosciences (PPGBio), Federal University of Health Sciences of Porto Alegre, Porto Alegre, Brazil; 6 Department of Parasitology, University of South Bohemia, Ceske Budejovice, Czech Republic; 7 Present Address: Department of Biology, University of Toronto Mississauga, Mississauga, ON L5L 1C6 Canada

**Keywords:** Physiology, Zoology

## Abstract

Juvenile hormone (JH) signalling, via its receptor Methoprene-tolerant (Met), controls metamorphosis and reproduction in insects. Met belongs to a superfamily of transcription factors containing the basic Helix Loop Helix (bHLH) and Per Arnt Sim (PAS) domains. Since its discovery in 1986, Met has been characterized in several insect species. However, in spite of the importance as vectors of Chagas disease, our knowledge on the role of Met in JH signalling in Triatominae is limited. In this study, we cloned and sequenced the *Dipetalogaster maxima Met* transcript (*DmaxMet*). Molecular modelling was used to build the structure of Met and identify the JH binding site. To further understand the role of the JH receptor during oogenesis, transcript levels were evaluated in two main target organs of JH, fat body and ovary. Functional studies using *Met* RNAi revealed significant decreases of transcripts for vitellogenin (*Vg*) and lipophorin (*Lp*), as well as their receptors. Lp and Vg protein amounts in fat body, as well as Vg in hemolymph were also decreased, and ovarian development was impaired. Overall, these studies provide additional molecular insights on the roles of JH signalling in oogenesis in Triatominae; and therefore are relevant for the epidemiology of Chagas´ disease.

## Introduction

Juvenile hormones (JHs) are sesquiterpenoids synthesized by the *corpora allata* (CA) playing critical roles during insect development and reproduction^1^. Sir Vincent B. Wigglesworth originally described JH in the Triatominae kissing bug *Rhodnius prolixus* as a “metamorphosis inhibitory hormone”, as well as a “yolk-forming hormone”^2–4^. It has been postulated that in the evolution of insects, the original function of JH was to control reproduction, and only later it was co-opted as a hormone regulating metamorphosis^5^.

Methoprene-tolerant (Met), the intracellular receptor for JH, is a ligand-activated member of the basic-Helix-Loop-Helix (bHLH)/Per-Arnt-Sim (PAS) protein family^6^. It has a modular architecture with DNA-binding, ligand-binding, dimerization, and transcriptional activation domains^7,8^. An active juvenile hormone receptor (JHR) comprises two bHLH‐PAS proteins, the JH-binding Met and its partner Taiman (Tai)^7,8^. Met is activated by binding of JH to the C-terminal PAS domain (PAS-B)^7^, causing the formation of a JH receptor complex with Tai^7,9^. The activated JHR dimer binds DNA at distinct JH response elements (JHREs), and triggers transcription of JH-regulated genes^10,11^. Met has an adaptable hydrophobic ligand-binding pocket that can accommodate methyl farnesoate (MF) and the different epoxidated JHs^9^, including JH III skipped bisepoxide (JHSB_3_), the sesquiterpene homolog present in kissing bugs^12,13^.

*Dipetalogaster maxima*, the largest of all Triatomine species, is a model organism for studying the reproductive physiology of kissing bugs^14–18^. Circulating JHSB_3_ levels during the reproductive cycle were recently reported in *D. maxima* females, with a sharp increase observed at day 4 after a blood meal^19^. We have also previously described that JH is critical for lipid storage in oocytes. It regulates vitellogenin (*Vg*) and lipophorin (*Lp*) gene expression in the fat body, as well as modulates the expression of the *Vg* and *Lp* receptor genes (*VgR* and *LpR,* respectively) in ovaries^19^.

In this work, two *Met* transcripts of *D. maxima* (*DmaxMet*) were cloned and sequenced, enabling their characterization, as well as in silico receptor-ligand interaction studies by bioinformatics analyses. To gain further insight into the role of JH signalling regulating oogenesis, we measured the changes in *Met* transcripts in the fat body and ovaries during the gonadotrophic cycle. In addition, we evaluated the effects of reducing the expression of *Met* by RNA interference on female reproductive phenotypes. Our results validated the important role of Methoprene-tolerant in JH-signalling regulating oogenesis in *D. maxima*.

## Results

### Sequence analysis and structural properties of DmaxMet

**Figure 1:**
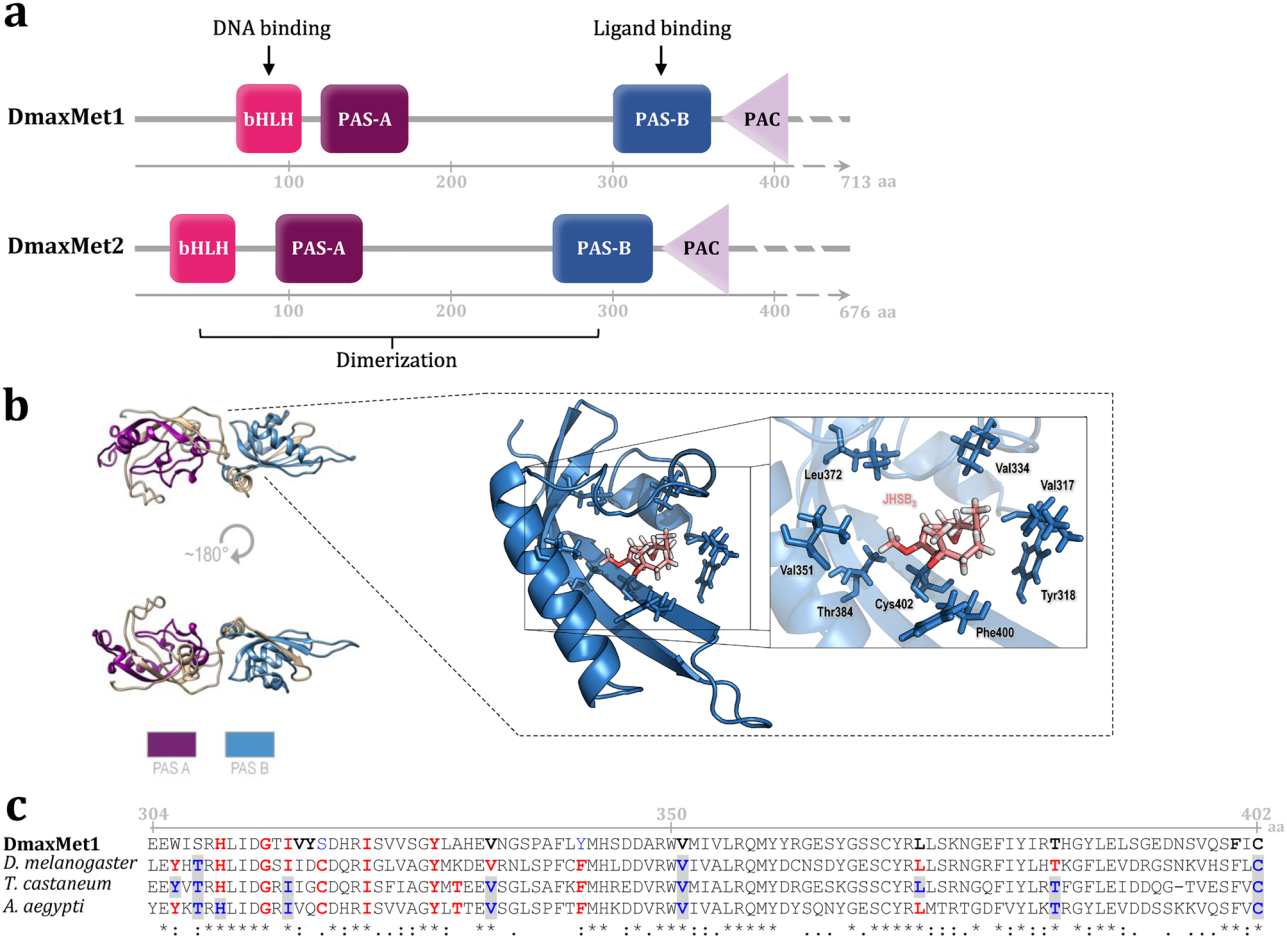
DmaxMet structure, modelling and docking. (**a**) Schematic view of DmaxMet1 (713 aa) and DmaxMet2 (676 aa) predicted protein sequences, showing the conserved domains, as well as DNA and ligand binding locations. (**b**) Structural model for DmaxMet1, displayed as a molecular surface superposed to a cartoon representation (left), with the detail of the in silico interactions between JHSB_3_ and the PAS-B domain of DmaxMet1 (right). (**c**) Alignment of DmaxMet1 PAS-B-PAC domains and *D. melanogaster* Met (NP_001285132.1), *T. castaneum* Met (NP_001092812.1), and *A. aegypti* Met (AAX55681.1), showing amino acid (aa) residues involved in JH-Met interactions. Gray line: amino acids (aa) position of DmaxMet; black bolded amino acids: aa affecting JH binding to DmaxMet1; shaded blue amino acids: mutations that affected JH binding to *D. melanogaster* Met*, T. castaneum* Met or *A. aegypti* Met; red amino acids: aa identified by docking or modelling as relevant for JH binding.

Using a Rapid Amplification cDNA Ends approach (RACEs) we cloned and sequenced the cDNA coding for Met in *D. maxima*. Two isoforms were found, *DmaxMet1* and *DmaxMet2* (accession numbers MZ361686.1 and MZ753950.1, respectively)*.* The *DmaxMet1* open reading frame (ORF) of 2456 nucleotides encodes a 713 amino acid protein (81 kDa) with an isoelectric point of 6.68. In addition, *DmaxMet2* presents an ORF of 2411 nucleotides, encoding a 676 amino acid protein (76 kDa) with an isoelectric point of 6.20 (Supplementary Fig. S1A). The deduced DmaxMet1 and DmaxMet2 proteins presented the classic domains reported for Met, namely, a bHLH DNA-binding domain, the tandemly arranged PAS-A and PAS-B domains and the PAS-associated C-terminal motif (PAC)^6,20^ (Fig. 1a and Supplementary Fig. S1B).

We employed the two *R. prolixus Met* isoforms sequences (*RpMet1* and *RpMet2*)^21^ to predict the molecular structures of DmaxMet1 and DmaxMet2 transcripts. *D. maxima* ORFs span 15 exons, with 13 of the exons showing high identity and similarity with their orthologs *RpMet1* and *RpMet2*. While *R. prolixus* transcript isoforms differ in the third exon sequences^21^, *D. maxima* transcripts vary in the first exon sequences. Phylogenetic analysis showed that the *Met* sequences of *D. maxima* and *R. prolixus* were arranged together, and did not cluster with those of the hemipterans *Nilaparvata lugens* and *Planococcus kraunhiae* (Supplementary Fig. S2).

Molecular modelling of DmaxMet was performed using the I-TASSER program^22^. Crystal structures for the PAS-B domains of Met have not been reported; consequently, our homology model was developed based on the crystal structure of the hypoxia-inducible factor 2α (HIF-2α), a member of the bHLH/PAS protein family^23^. Only the region of the full sequence containing both PAS domains was used to build the model (Fig. 1b). The TM-score (Template Modelling score) was 0.72 ± 0.11 (a TM score > 0.5 indicates correct topology)^22^.

Because the crystallized PAS region is similar in both DmaxMet isoforms, the three-dimensional protein structure obtained from DmaxMet1 was used for in silico docking of JHSB_3_. The ligand docking exposed the amino acids bordering an inferred hydrophobic JH-ligand pocket in the PAS-B domain (Fig. 1b). Alignment of PAS-B domains from DmaxMet1, *Tribolium castaneum* Met, *Aedes aegypti* Met and *Drosophila melanogaster* Met highlighted the conserved and mutated amino acid residues that are relevant for JH-binding identified by either modelling or mutagenesis experiments in these four species (Fig. 1c).

### Changes in *DmaxMet* mRNA levels throughout the reproductive cycle of *D. maxima*

**Figure 2:**
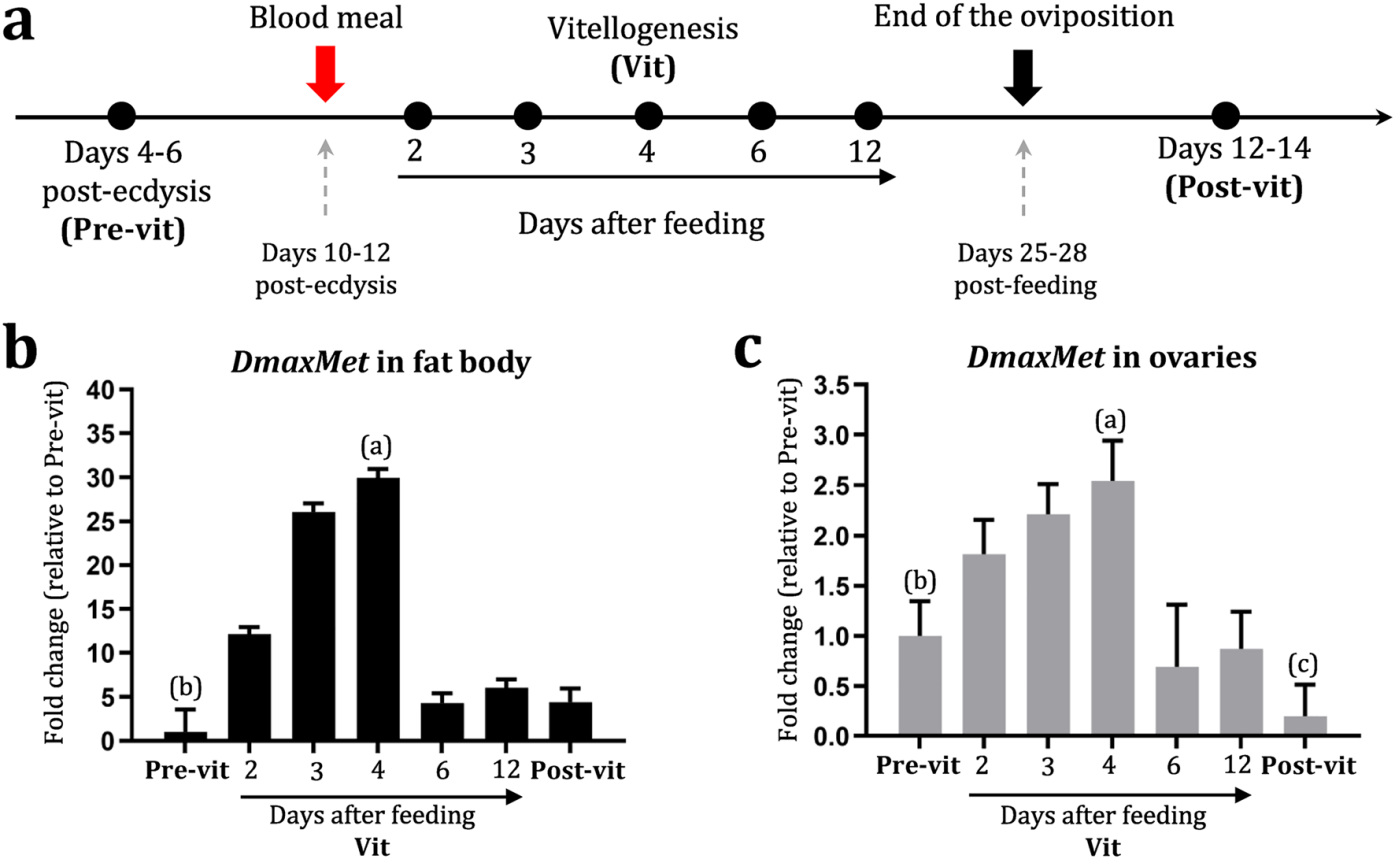
mRNA temporal profiles of *DmaxMet* throughout the reproductive cycle. (**a**) Experimental scheme showing the timeline of sampling days. (**b**) Temporal expression of *DmaxMet* in fat body (*P* < 0.05 vs. Pre-vit; 2, 6, 12 days after feeding and Post-vit; *P* < 0.005 vs. 2, 3, 4 days after feeding). (**c**) Temporal expression of *DmaxMet* in ovaries (*P* < 0.05 vs. 6 and 12 days after feeding and Post-vit; *P* < 0.01 vs. 2, 3, 4 days post feed; *P* < 0.05 vs. Pre-vit). The results are shown as mean ± SD of three independent experiments. Each experimental point includes samples from insects processed individually (n = 3). The statistical analyses were conducted using parametric one-way ANOVA and the post test was performed using Student–Newman–Keuls multiple-comparisons.

Transcript levels of the *DmaxMet* gene were analyzed in the fat body and ovaries at representative days during the reproductive cycle (Fig. 2a). The results showed that in both tissues, mRNA levels were low during pre-vitellogenesis, increased at the beginning of vitellogenesis, and reached maximum levels at day 4 after the blood meal. Afterward, *DmaxMet* transcripts sharply dropped as vitellogenesis progressed, and by day 6 after feeding, levels were low and similar to those in pre-vitellogenic and post-vitellogenic females (Fig. 2b,c).

### Effect of *DmaxMet* gene silencing

**Figure 3:**
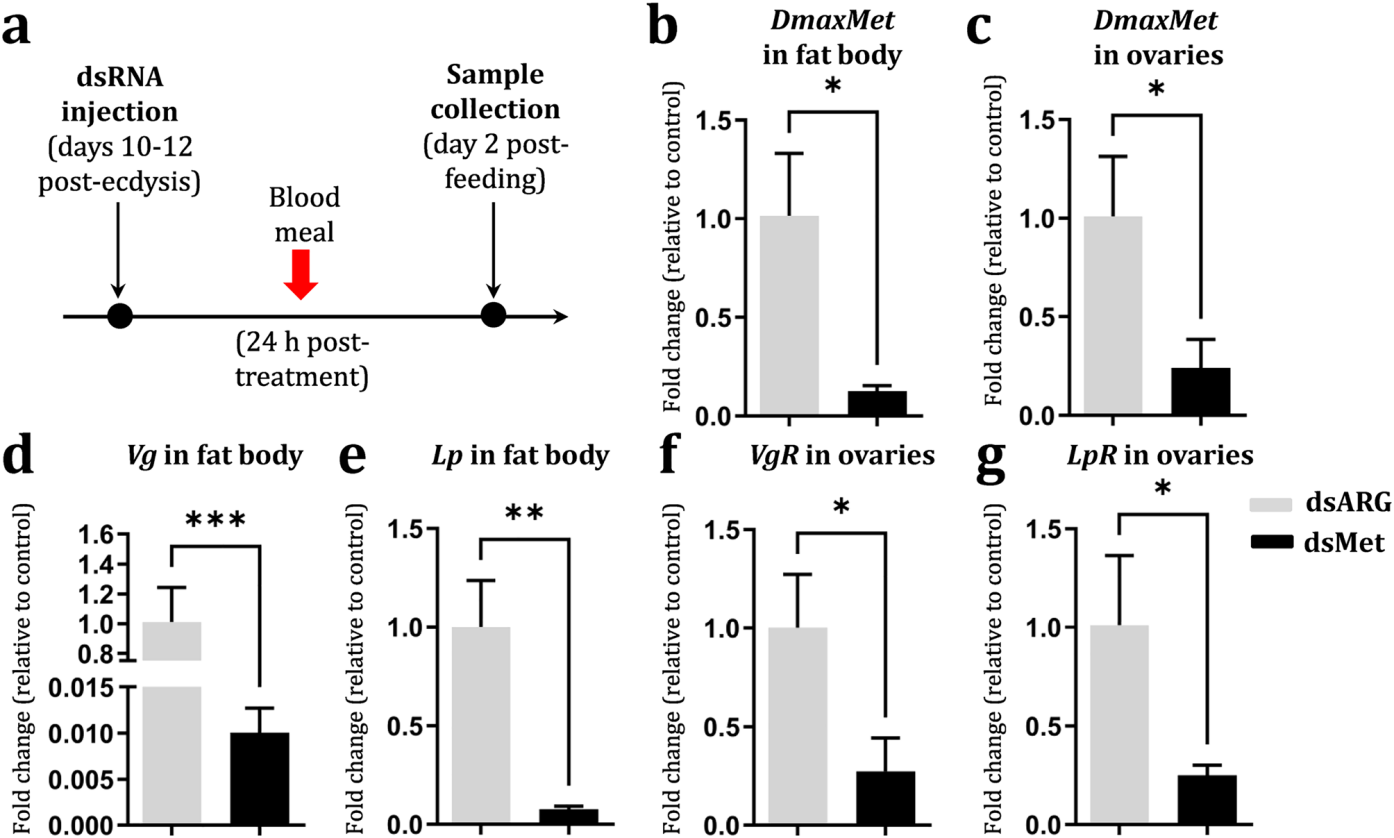
Effect of *DmaxMet* gene silencing. (**a**) Experimental scheme. (**b**–**c**) Knockdown efficiency of dsMet treatment in the fat body (**b**) and in ovaries (**c**). (**d**–**e**) Effect of dsMet and dsARG (control) treatment on *Vg* (**d**) and *Lp* (**e**) transcript expression in the fat body. Effect of dsMet and dsARG treatment on *VgR* (**f**) and *LpR* (**g**) transcript expression in ovaries. The statistical analyses were performed using Student’s t-test. The results are shown as mean ± SD of three independent experiments and included samples from insects processed individually (*n* = 3). *P < 0.05; **P < 0.01; ***P < 0.001.

To better understand the role of Met in the regulation of oogenesis, we performed the knockdown of *DmaxMet* gene expression by RNAi in pre-vitellogenic females (Fig. 3a). Survival rates and amounts of blood ingested showed no significant differences between dsDmaxMet-treated insects and the control insects injected with a dsRNA molecule encoding the partial sequence of ampicillin resistance gene (dsARG). The efficiency of gene knockdown, evaluated by qPCR, indicated that *DmaxMet* transcript levels at day 2 after RNAi treatment were reduced by 84% in fat body and by 78% in ovaries (Fig. 3b,c).

**Figure 4:**
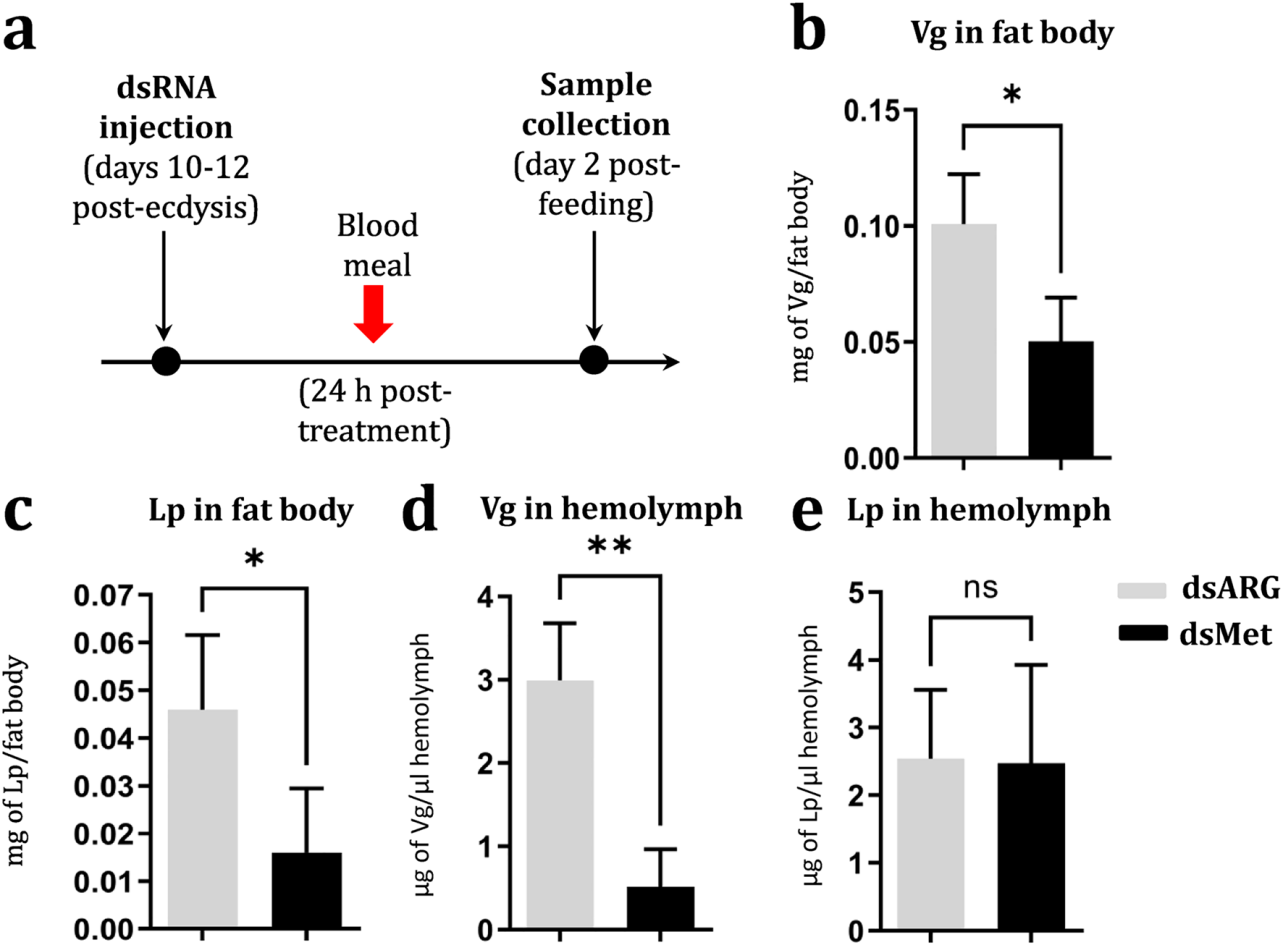
Effect of *DmaxMet* gene silencing on vitellogenin and lipophorin protein levels. (**a**) Experimental scheme. Effect of dsMet and dsARG (control) treatment on Vg (**b**) and Lp (**c**) in the fat body. Effect of dsMet and dsARG treatment on Vg (**d**) and Lp (**e**) levels in hemolymph. The results are shown as mean ± SD of three independent experiments and included samples from insects processed individually (*n* = 3). *P < 0.05; **P < 0.01.

Knockdown of the *DmaxMet* gene caused significant decreases of *Vg* and *Lp* mRNA levels in the fat body (Fig. 3d,e), as well as *VgR* and *LpR* transcripts in ovarian tissue (Fig. 3f,g). At the protein level, the contents of Vg and Lp in fat body notably decreased in *DmaxMet*-silenced females (Fig. 4a–c). However, when assessed in the hemolymph, Vg levels were severely reduced in RNAi-treated females (Fig. 4d), whereas no differences on Lp titers were observed between dsDmaxMet and dsARG injected insects (Fig. 4e).

**Figure 5:**
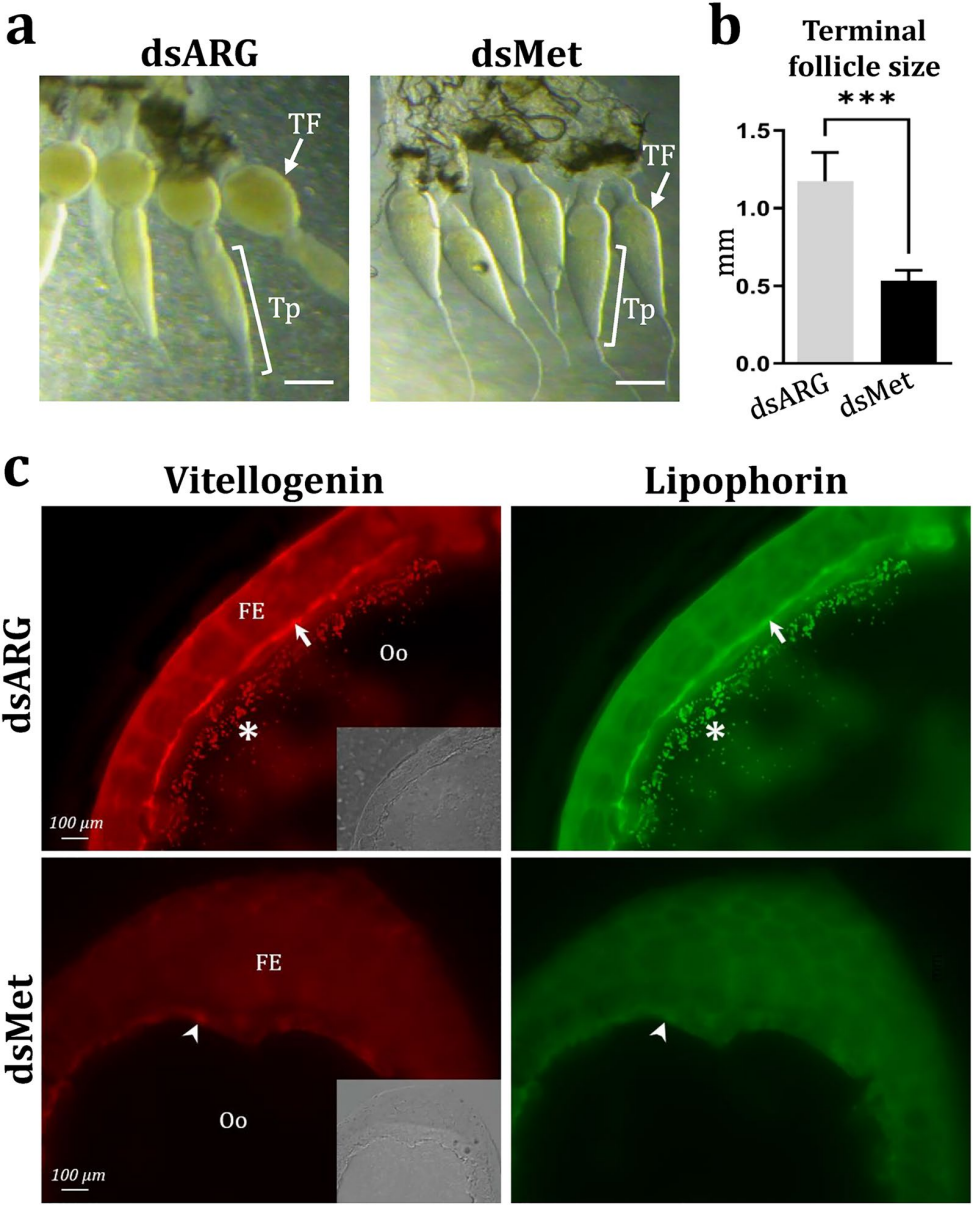
Effect of *DmaxMet* gene silencing on ovarian development. (**a**) Representative ovarian tissue showing terminal follicles from insects treated with either dsARG (control) or dsMet, two days after blood meal. (**b**) Terminal follicle size from dsRNA-treated insects. The results are shown as mean ± SD of three independent experiments and included samples from insects processed individually (*n* = 4). The statistical analyses were performed using Student’s t-test (***P < 0.001). (**c**) Effect of dsMet and dsARG treatment on Vg and Lp uptake by developing oocytes. Positive signal for Vg (red) and Lp (green) observed in the perioocytic space and oocyte membrane (arrows) as well as in the oocytes (asterisks) of dsARG-treated females (controls) was decreased or faintly detected in dsMet-silenced insects. Bars: 100 µm. FE (follicular epithelium); TF (terminal follicle); Tp (tropharium). Similar results were obtained in three independent experiments.

*DmaxMet* gene silencing also influenced the maturity of ovarian follicles, radically decreasing the development of terminal or mature follicles (Fig. 5a,b). When analyzed by immunofluorescence, the signals for Vg and Lp were notably decreased in the perioocytic space and oocyte membrane, and weakly detected in the oocytes of terminal follicles in *DmaxMet*-silenced females compared to controls; consistent with an impairment of lipoprotein uptake by the developing oocytes (Fig. 5c).

## Discussion

In adult female Triatominae, a blood meal triggers a sequence of physiological processes controlled by endocrine factors and leading to a successful completion of oogenesis^24^. Almost a century ago, Sir Vincent B. Wigglesworth described that females of *R. prolixus* decapitated 24 h after feeding arrested ovarian development^3^. Later, it was reported that the CA was the source of JH, the main hormone controlling reproduction in Triatominae^25^. Since then, the effects of JH and its signalling pathway have been extensively studied. However, for many decades, attempts to identify the JH receptor were unsuccessful. Methoprene is a potent analogue of JH used as an insecticide^26^. In the 1980s it was described a *D. melanogaster* mutant line resistant to the lethal effects of Methoprene. The mutated gene was essential for the action of the JH analogue, and it was named *Met*, for Methoprene tolerant^27^. The role of Met as the intracellular receptor for JH has been validated by multiple studies in holometabolous and hemimetabolous insects^20^. A previous study performed a genomic and functional characterization of *Met* in *R. prolixus* (*RpMet*)^21^. Silencing of *RpMet* resulted in precocious metamorphosis and abnormal ovarian development^21^. Precocious development of adult features in *R. prolixus* nymphs were also reported upon silencing of Krüppel-homolog 1 (*Kr-h1*), a JH-inducible repressor of metamorphosis^28^.

We cloned and sequenced the transcripts coding for *Met* in *D. maxima*. A phylogenetic analysis showed that the *DmaxMet* translated nucleotide sequences are evolutionarily related to *RpMet,* encoding also two isoforms^21^. Both DmaxMet isoforms present the bHLH, PAS-A and PAS-B domains described in other insect species, supporting the statement that the JH receptor is highly conserved^1,7,20^.

Our modelling and ligand docking revealed amino acids bordering an inferred hydrophobic JH-ligand pocket in the PAS-B domain. As depicted in Fig. 1c, most of these amino acid residues are conserved in Met of other insect species, and have been identified as critical for JH-binding by modelling and mutagenesis experiments. In *T. castaneum*, mutations T254Y, V280F, V297F, T330Y, 347 M, I262F and L318F hindered JH III binding^7^. In *A. aegypti*, 14 residues were predicted to be important to bind JH-III: T403, H405, I411, I418, V429, F437, V446, L450, S463, Y465, L467, T479, C497 and N499; while JH-III binding was experimentally hampered by T403Y, V429F, T479Y, C497M, H405R, I411F and V446F mutations^29^. In *D. melanogaster*, mutations of T272Y, V315F, and C366M abolished JH binding^30^. Our docking studies using JHSB_3_ revealed that similar conserved amino acids bordered an inferred hydrophobic JH-ligand pocket in the PAS-B domain of *D*. *maxima*. Interestingly, while docking model of Met-JH III complex in *T. castaneum* predicted a hydrogen bound between the hydroxyl group of Tyr-252 and the epoxide moiety of JH III^7^, the docking of JHSB_3_-DmaxMet only established hydrophobic interactions.

Triatominae such as *D. maxima* and *R. prolixus* have JHSB_3_, a doubled-epoxidated form also present in other heteropteran species^12,13,31^. Circulating JHSB_3_ levels during the reproductive cycle were recently reported in *D. maxima* females, with a sharp increase observed at day 4 after a blood meal^19^. We now established that *DmaxMet* mRNA expression in fat body and ovaries showed maximum levels of expression at 4 days post-feeding, when JHSB_3_ hemolymph titers are high.

Insect vitellogenesis is regulated by JH and 20-hydroxyecdysone (20E). JH stimulates vitellogenesis in hemimetabolous and most holometabolous insects. 20E contols vitellogenesis in selected dipterans, lepidopterans and hymenopterans (reviewed by Wu et al., 2021)^32^**.** Recent reviews have covered the pleiotropic functions of JH in female insect reproduction^1,33^. Topical application of JH III on pre-vitellogenic *D. maxima* females stimulated the transcription of *Vg* and *Lp* genes in the fat body; while females treated with the anti-JH compound Precocene II exhibited a down-regulation in the transcription of *Vg*^19^. Our results using *DmaxMet* RNAi confirmed that the JH-Met pathway plays a critical role in the regulation of *Vg* expression in Triatominae^24^. Remarkably, knockdown of *DmaxMet* gene also down-regulated the *L*p gene in the fat body, although it did not impact the Lp levels in the hemolymph. It is known that in addition to the rate of synthesis in the fat body, circulating Lp is influenced by other factors, such as its long half-life that favors the cycling among tissues, to exchange lipids as a reusable shuttle^34^.

Previous studies highlighted the role of JH controlling yolk incorporation into developing oocytes in insects; JH is involved in the transcriptional regulation of *VgR* in the red ant *Solenopsis invicta*^35^, as well as in *VgR* and *LpR* expression in *Ae. aegypti*^36^. In addition, it has been reported a post-transcriptional control of *LpR* during the reproductive cycle of the cockroach *Blatella germanica*^37^. In the cockroach *Diploptera punctata*, Met-silenced insects showed impaired vitellin storage in the terminal follicles^38^. In the oocytes of two cockroach species, vitellogenesis induced changes in the subcellular distribution of VgR and LpR, which translocated to the cortical zone to facilitate the receptor-ligand interaction and further lipoprotein uptake^39^. In Triatominae, knowledge of the role of JH on the expression of these receptors is limited.

In *D. maxima*, we previously described the action of JH on the expression of *VgR* and *LpR* genes and on their ligands Vg and Lp^19^. In the current studies we report that *DmaxMet* gene silencing resulted in decreased *VgR* and *LpR* transcript levels, and therefore impaired Vg and Lp uptake by oocytes, with the associated reduction in the development of terminal follicles.

## Conclusion

We characterized two *Met* transcripts in *D. maxima*. Modelling and ligand docking revealed conserved critical amino acids in the PAS-B domain JH-ligand pocket. Changes in *Met* transcript levels during the oogenesis temporally coincided with JH titer fluctuations^19^. *DmaxMet* silencing decreased *Vg* and *Lp* transcript levels. Silencing of *Met* gene also induced the down-regulation of *VgR* and *LpR* genes, and severely compromised ovarian development. Our present work provides additional compelling evidence that JH-Met signaling is critical for oogenesis in female Triatominae, offering novel perspectives for the control of Chagas´ disease vectors.

## Methods

### Ethics statement

Housing conditions and manipulation of hens employed in the maintenance of the insect colony followed the protocol authorized by the Animal Care Committee of the Centro de Investigaciones en Bioquímica Clínica e Inmunología (CIBICI-CONICET-Universidad Nacional de Córdoba). They followed the guidelines published by the Canadian Council on Animal Care with the assurance number A5802-01 delivered by the Office of Laboratory Animal Welfare (National Institutes of Health). The animal facility at the CIBICI-CONICET is a dependency of the Argentine National Ministry of Science Science (http://www.bioterios.mincyt.gob.ar). No infected insect species, human blood, or hen sacrifices were involved in the study. Details of the approved protocol were previously published^17^.

### Insects

Anautogenous females were taken from a colony of *D. maxima*, maintained at 28 °C, 70% relative humidity, 8:16 h light/dark photoperiod. Standardized conditions of insect rearing were previously described^15^. Briefly, females were separated from males at the last nymphal stage before blood feeding. Newly emerged females were segregated individually and placed in jars together with two fed males. Successful mating was confirmed by observation of the spermatophore. Mated females were kept in individual jars until they were able to take a blood meal (days 10–12 post-ecdysis), which resulted in a 3.0–5.5-fold increase in the body weight of the insect. Thereafter, egg laying was daily monitored, and the beginning and the end of the oviposition period were recorded. Unless otherwise stated, experiments were performed by sampling hemolymph and tissues at representative days of the reproductive cycle as previously descibed^15,17^: pre-vitellogenesis (days 4–6 post-ecdysis, unfed period); vitellogenesis (days 2, 3, 4, 6 and 12 post-blood feeding); and post-vitellogenesis (days 12–14 after the end of oviposition).

### Hemolymph and tissues sampling

Hemolymph was individually collected from immobilized females with a Hamilton syringe by cutting off the legs at the level of the coxa and gently pressing the abdomen. The collected samples were placed in cold tubes containing 10 mM Na_2_EDTA, 5 mM dithiothreitol (DTT) and a cocktail of protease inhibitors (P8340, Sigma-Aldrich, St. Louis, MO, USA) as previously described ^17,19^. Hemolymph samples were centrifuged at 10,000 × *g* for 10 min at 4 ℃ to remove hemocytes. Protein amounts were measured using the Bradford method^40^. Samples were stored at − 70 °C until use. Ovarian tissues and fat bodies from females at representative days of the reproductive cycle were dissected out in cold phosphate-buffered saline (PBS: 6.6 mM Na_2_HPO_4_/KH_2_PO_4_, 150 mM NaCl, pH 7.4), using a standard stereoscope, as previously described ^17,19^.

### Cloning the full-length cDNA of *DmaxMet*

The complete sequences of the *DmaxMet* transcripts were obtained by Rapid Amplification cDNA End (RACE) (SMARTer RACE 5′/3′ kit, Takara Bio USA, Inc). Briefly, for the first cDNA synthesis step, a universal mixture of primers was used to recognize specific regions of the 3′ or 5′ ends (3′ RACE-cDNA or 5′ RACE-cDNA, respectively). Synthesis of RACE-cDNAs was performed using 1 μg of ovarian total RNA. For the RACE amplification reaction, *DmaxMet* gene-specific primers were: a forward primer for 3′ RACE-cDNA and a reverse primer for 5′ RACE-cDNA. To achieve optimal yields, the *DmaxMet* primers were designed with a length of 23–28 nucleotides (nt), containing a guanidine/cytosine (GC) ratio of 50–70% and a melting temperature (Tm) > 70%. Additionally, a 15 nt sequence (GATTACGCCAAGCTT), necessary for the insertion of the gene of interest into the *p*-RACE vector, was added to the primers. Once the amplification reaction was completed, the size of the RACE product was verified by 1% agarose gel electrophoresis. For optimal quality in the sequencing, the fragments obtained by RACE were cloned on the *p*-RACE vector following the manufacturers' recommendations. The clones obtained were then sequenced using a 3130XL Genetic Analysers in the DNA core facility at FIU (Miami, FL, USA).

### Bioinformatic analyses and modelling

The deduced amino acid sequence, molecular mass and isoelectric point prediction for the two *DmaxMet* isoforms were assessed using tools available on Expasy (http://web.www.expasy.org; SIB Bioinformatics Resource Portal). The Basic Local Alignment Search Tool algorithm (BLAST, https://blast.ncbi.nlm.nih.gov/Blast.cgi) was employed to compare the sequence of the transcripts coding for *DmaxMet* isoforms with other *Met* sequences deposited in databases. BLAST was also used to predict the major functional domains of the receptor. Mapping of exons in *DmaxMet* was achieved with the Exon–Intron Graphic Maker (http://wormweb.org/exonintron), taking as references the homologous sequences reported for *R. prolixus*^21^.

The molecular model of DmaxMet was built by homology modelling using I-TASSER package^22^. The HIF2α–Arnt crystal structure (PDB ID 3F1P) was chosen as an homologous template^7^. The visualization of the structures was performed by UCSF Chimera^41^ and PyMOL (The PyMOL Molecular Graphics System, Version 2.0 Schrödinger, LLC).

The obtained DmaxMet three-dimensional model was used in docking simulation to analyze the interaction between DmaxMet and JHSB_3_, only the DmaxMet PAS domain were considered, since they are the only domains with crystallized templates deposited in databases. The docking calculations were carried out with DockThor version 2^42,43^.

For the evolutionary analysis of Met, the predicted amino acid sequences for *DmaxMet* were compared with selected Met sequences retrieved from the NCBI database. The sequences were aligned using MAFFT^44^, and twenty alternative full-length alignments were combined via GUIDANCE2^45^ and used for inferring the protein phylogeny. The phylogenetic tree was constructed with PhyML^46^ under Maximum Likelihood model (LG + G + I)^47–49^, using branch support analysis^50^.

### RNA extraction and reverse transcriptase quantitative real-time PCR (RT-qPCR)

Ovaries and fat bodies were subjected to RNA extraction with TRIzol (Thermofisher Waltham, MA, USA), according to the manufacturer's instructions. Samples were treated with DNase to remove genomic DNA (Promega, Heidelberg, Germany) and RNA integrity was evaluated by 1% agarose gel electrophoresis. cDNA synthesis from 2 µg of total RNA was performed using the Moloney Murine Leukemia Virus reverse transcriptase protocol (M-MLV, Promega, Heidelberg, Germany). The qPCR assays were performed with an ABI Prism 7500 sequence detection system (Applied Biosystems, Foster City, CA, USA) using a Power SYBR Green PCR master mix (Thermo Fisher Scientific, Waltham, MA, USA), as previously described^19^. The 2^−ΔΔCt^ method was employed to evaluate of relative changes in gene expression^51^, using 18S ribosomal RNA (18S rRNA) as a reference gene ^19,52^. Unless otherwise stated, all reactions were carried out by triplicate. The primer sequences of all genes assayed are displayed on Supplementary Table 1. In the case of *DmaxMet*, the primers were designed to target a region shared by the *DmaxMet 1* and *DmaxMet 2*. In *D. maxima*, vitellogenin (Vg) is the product of the genes *Vg1* and *Vg2*, the latter showing the highest level of expression^19^. Therefore, in the present work *Vg2* was the gene quantified and named *Vg* for simplicity.

The amplification efficiency for each pair of primers was calculated using standard curves generated by serial dilutions of cDNA. All reactions showed an efficiency higher than 96% for the different pair of primers tested.

### Double-stranded RNA (dsRNA) synthesis and knockdown of *DmaxMet gene expression*

A 317 base pair *Met* template was used to synthesize a double-stranded RNA (dsRNA) molecule. Gene-specific primers targeting a region shared by the two *DmaxMet* isoforms were designed and then combined with the T7 RNA polymerase promoter sequence. The synthesis of dsRNAs was carried out using the MEGAScript RNAi kit (Thermo Fisher Scientific), according to the manufacturer’s recommendations. dsRNAs were precipitated with ammonium acetate/ethanol, and then suspended in ultrapure RNase-free water to a final concentration of 5 μg/μl. A dsRNA molecule based on the ampicillin resistance gene (dsARG) from the pGEM-T Easy Vector system was used as a control, as previously described ^19,53^.

To knockdown the expression of *DmaxMet*, adult females at 10–12 days post-ecdysis were injected into the hemocoel with 10 µg of dsMet or dsARG (control) dissolved in 2 µl of ultrapure water using a Hamilton microsyringe. At 24 h post-injection, dsRNA-treated insects were fed with blood, and 2 days later fat bodies and ovaries were individually sampled and processed by qPCR to determine the *DmaxMet* mRNA levels. The effect of *DmaxMet* silencing on *Vg* and *Lp* gene expression in fat bodies as well as on *VgR* and *LpR* in ovarian tissue was also assessed by qPCR.

### Vitellogenin and lipophorin assessments

To further evaluate the relevance of *DmaxMet* knockdown on the yolk protein precursors Vg and Lp metabolism, a group of females were treated with dsMet or dsARG (control). At 24 h post-injection, dsRNA-treated insects were fed with blood, and 2 days later hemolymph, fat bodies and ovaries were collected. Quantification of Vg and Lp was accomplished by indirect enzyme-linked immunosorbent assay (ELISA), using a polyclonal anti-vitellin antibody (anti-Vt, 1:4000), and a polyclonal anti-lipophorin antibody (anti-Lp, 1:5000) as previously reported^15,19,54^. Purified Vt and Lp were employed to build calibration curves^14,54^. For the assays, the hemolymph was individually collected and directly processed for ELISA. Fat bodies were weighed and then homogenized in buffer Tris-NaCl (20 mM Tris, 150 mM NaCl, pH 7.4) containing a cocktail of protease inhibitors. The homogenates were first centrifuged at 2500 × *g* for 10 min at 4 °C and the floating fat cake and pellet were discarded. The resulting material was centrifuged at 15,000 × *g* for 30 min at 4 °C and the supernatants collected and used for ELISA, as previously described^19^.

Vg and Lp were also evaluated in ovarian tissue by immunofluorescence assays, as previously described^19^. Briefly, the ovaries from *dsDmaxMet* or control females were dissected out and fixed with 4% paraformaldehyde in PBS, transferred to PBS/sucrose and processed for cryostat sectioning^54^. Thereafter, tissue sections (10 µm) were obtained using a Leica CM1510 cryostat (Leica Microsystems, Wetzlar, Germany) and placed onto poly-L-lysine-treated glass slides (Sigma-Aldrich, St. Louis, MO, USA). For the assays, the slides were blocked with 1% BSA, 0.1% Triton X-100, 5% fetal bovine serum in PBS during 1 h, and then, incubated sequentially as follow: anti-Vt antibody (1:4000), followed by a secondary anti-rabbit IgG antibody labeled with Alexa Fluor 594 (Molecular Probes, Carlsbad, CA, USA); anti-lipophorin antibody labeled with FITC (anti-Lp-FITC, 1:10). Slides were mounted in Fluorsave (Calbiochem, Darmstadt, Germany) and examined in a Leica DMi8 microscope (Leica Microsystems, Wetzlar, Germany).

### Statistical analysis

The results are presented as the mean ± Standard Deviation (SD) from at least three independent experiments as stated in the legend to figures. Each experimental point includes ovaries, fat body or hemolymph from insects processed individually (*n* = 3–4). Graphs and statistical tests were performed using GraphPad Prism 6.0 and GraphPad Instat 3.0. (GraphPad Software, San Diego, CA, USA). The data were evaluated for normality and homogeneity of variance using the Shapiro–Wilk test, which showed that no transformations were needed. All datasets passed normality and homoscedasticity tests. Statistical analyses were performed employing Student's t-test or parametric one-way ANOVA, and using the Student–Newman–Keuls multiple-comparisons as a post-test. A *P* value < 0.05 was considered statistically significant.

## Supplementary Information

Supplementary Information 1.

Supplementary Information 2.

## Data Availability

All data generated or analysed during this study are included in this published article and its supplementary information files. The sequences of two new isoforms were deposited in the NCBI database. https://www.ncbi.nlm.nih.gov/search/ DmaxMet1 (accession number MZ753950) and DmaxMet2 (accession numberMZ753950).
