# Supplementary material for: Role of Methoprene-tolerant in the regulation of oogenesis in Dipetalogaster maxima
Source: Sci Rep. 2022 Aug 20;12:14195. doi: 10.1038/s41598-022-18384-5 (PMC9392760; doi:10.1038/s41598-022-18384-5)
Supplement: Supplementary file 1 — Supplementary Information 1. [file 41598_2022_18384_MOESM1_ESM.pdf]

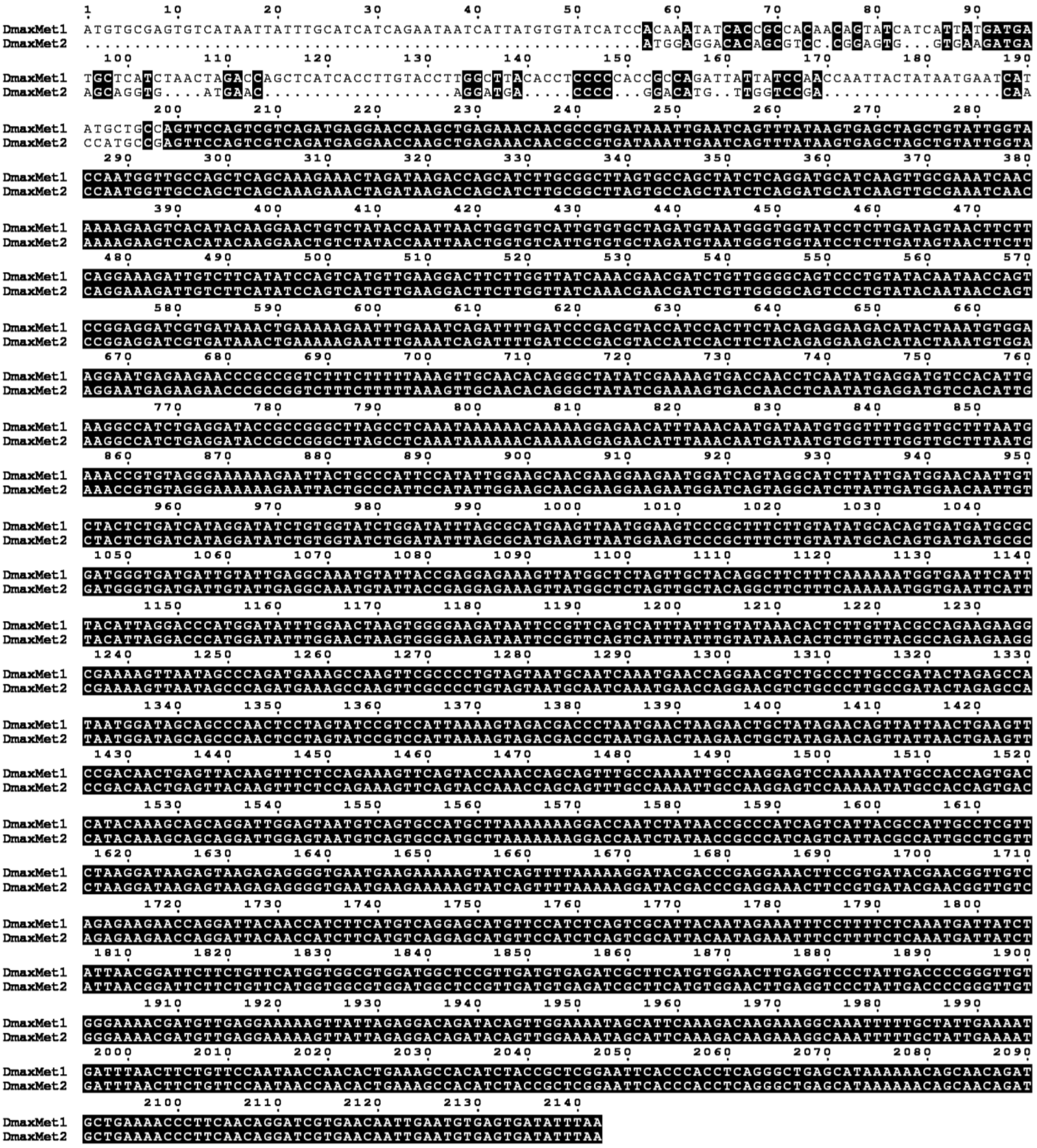

Supplementary Figure 1A. Alignment between *DmaxMet1* and *DmaxMet2* nucleotide sequences.

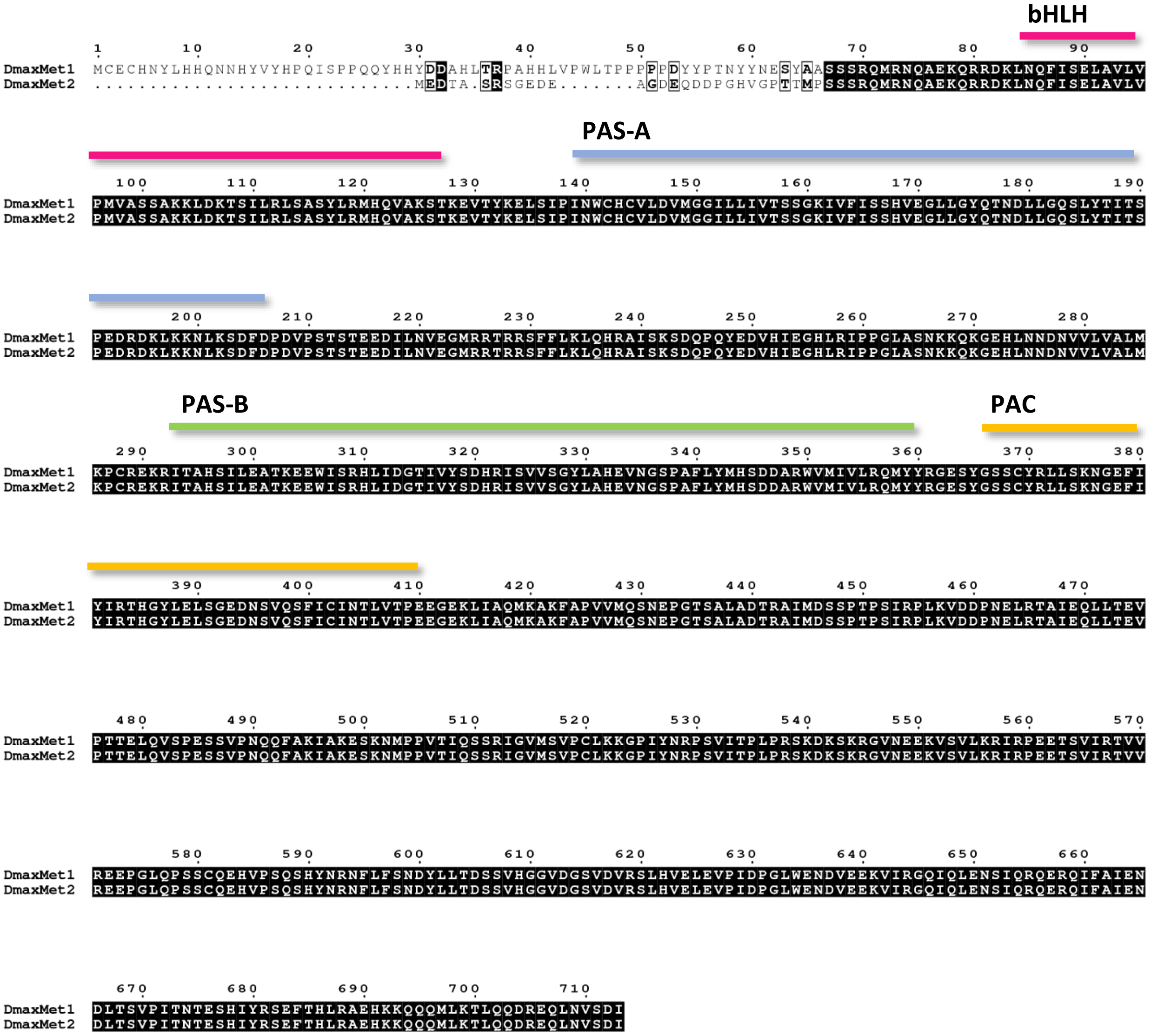

Supplementary Figure 1B. Alignment between DmaxMet1 and DmaxMet2 amino acid sequences and common domains: bHLH (fuchsia), PAS-A (blue), PAS-B (green) and PAC (yellow).

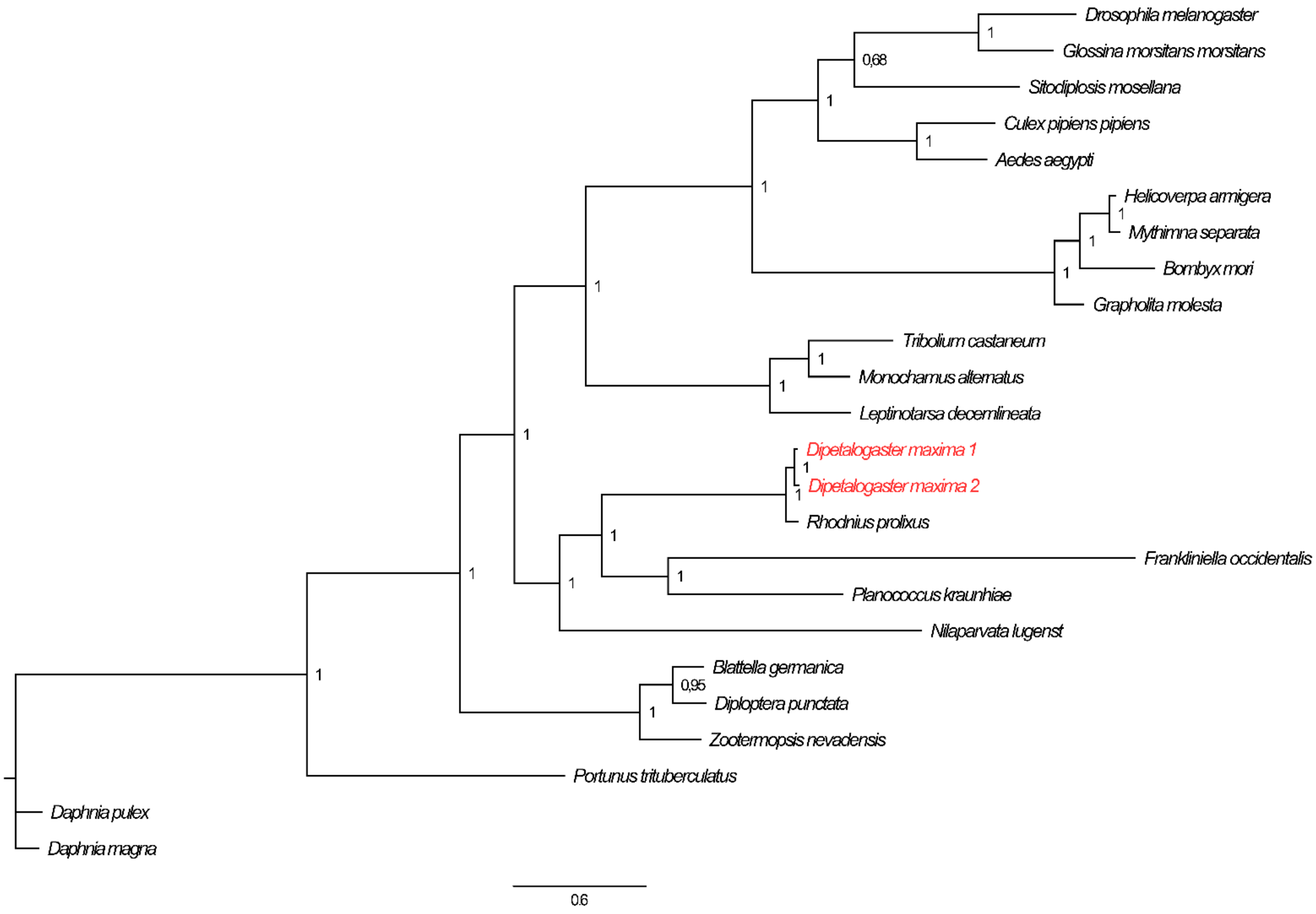

Supplementary Figure 2. Phylogram of selected invertebrate MET sequences. The phylogeny was reconstructed under Maximum Likelihood and VT+G model while branch support was estimated by aLRT.
