# Supplementary material for: Role of Methoprene-tolerant in the regulation of oogenesis in Dipetalogaster maxima
Source: Sci Rep. 2022 Aug 20;12:14195. doi: 10.1038/s41598-022-18384-5 (PMC9392760; doi:10.1038/s41598-022-18384-5)
Supplement: Supplementary file 2 — Supplementary Information 2. [file 41598_2022_18384_MOESM2_ESM.docx]

| Genes | Primers | Sequence (5’---3’) |
| --- | --- | --- |
| *18s Ribosomal RNA* | Forward | TCGGCCAACAAAAGTACACA |
|  | Reverse | TGTCGGTGTAACTGGCATGT |
| *VgR* | Forward | CATCTCTTGGTATGTTACACT |
|  | Reverse | CCTGACTGCAAGGACGGTTC |
| *Vg1* | Forward | ACTACTGACTACAACCACTG |
|  | Reverse | TAACGGAAGAGCGTGCAAGGA |
| *LpR* | Forward | GCCACACGATATGTTCGATG |
|  | Reverse | GGTCTTCCGTTGTCTTCCTG |
| *Lp* | Forward | CTGTACTCAAGGAATCTGGAT |
|  | Reverse | TTGATTGAGATGTTCCTGTA |
| *Met* | Forward | AACTGGTGTCATTGTGTGCTAGA |
|  | Reverse | ATGGTACGTCGGGATCAAAA |
| RNAi construct | **Primers** | **Sequence (5’---3’)** |
| T7-dsARG | Forward | **taatacgactcactatagggaga**ATGAGTATTCAACATTTCCGTGTC |
|  | Reverse | **taatacgactcactatagggaga**AATAGTTTGCGCAACGTTG |
| T7-dsMet | Forward | **taatacgactcactatagggaga**AACTGGTGTCATTGTGTGCTAGA |
|  | Reverse | **taatacgactcactatagggaga**ATGGTACGTCGGGATCAAAA |
| RACE | **Primers** | **Sequence (5’---3’)** |
|  | Forward | gattacgccaagcttGATCTGTTGGGGCAGTCCCTGTAT |
|  | Reverse | gattacgccaagcttAGTGGATGGTACGTCGGGATCAAA |

**Supplementary Table 1.** The primer sequences.

***taatacgactcactatagggaga** = T7 RNA polymerase promotor.
